# Letter to the Editor

**DOI:** 10.1590/S0102-67202014000200019

**Published:** 2014

**Authors:** Pedro Luiz Squilacci Leme

Reading the Letter to the Editor written by Professor Fabio Gonçalves Ferreira, São Paulo titled "The ABCD indexing in PubMed and surgery of schistosomiasis with portal hypertension in Brazil" (Arq Bras Cir Dig 2013; 26 (3): 248-251)^1^ I would also like to congratulate the editorial board for his recent promotion in Qualis indexation, in the area of Medicine III, when it was raised to B3.

I consider it necessary to congratulate the Editor for the option to publish the articles also in English, greatly increasing the reach and impact of the publication, as well as the speed, the less bureaucratic and friendly way to contact the journal and even the Editor. To illustrate the quality of the journal reviewers, I emphasize a recent publication of ABCD: "Ultrasound aspects and anatomy of the aponeurosis of the transversus abdominis muscle" (Arq Bras Cir Dig 2013; 26 (3): 184-189)^2^, that received the ward of Best Poster of 29th International Congress of the Medical Women's International Association, held in Seoul, Korea, between July 31 and August 3, 2013.

When evaluating indexed journals available in our country, that accept articles on experimental surgery, we found a great lack of options. The research group I represent has articles reviewed by the Editorial Board of journals that are awaiting publication for periods next two years.

The old General Surgery, in the last decades of the last century, developed a new specialty, Digestive System Surgery, due to the increased complexity of the procedures performed on the digestive system, including organ transplants. Major surgery services currently provide places for two medical residency programs, surgery of the digestive system and the advanced general surgery, required by the complexity of current operations, which hinder the proficiency of a single surgeon in such specific operations.

During the graduation period in Medicine, the choice of medical specialty, that each student will develop during his professional life, begins to take shape, and students need to know all the basic specialties to face the evidence of access to medical residency. The Experimental Surgery at this stage shown an excellent teaching tool when developed as Scientific Initiation Program or University Extension, helping the Operative Technique academic discipline and awakening the interest in the study of basic subjects such as physiology and advanced as microsurgery^3^. Postgraduate in turn, also leads high-level specialists to research laboratory for the development of Master's dissertations and doctoral theses. Despite all these factors, the experimental paper have a lower weight for publications, since they are carried out on animals and their data are considered unsuitable for comparisons with pathophysiologic aspects of human beings, even with the recent concepts of Translational Medicine, looking to improve the interrelationship between the knowledge developed in the laboratories of several areas of Health Sciences with medical practice.

As for the ABCD, although specific focus on digestive surgery as determined by its title, accepts Experimental Surgery of articles, provided they are related to the digestive systemc^4^. I consider pertinent to suggest that in Journal's Editorial Board meeting be proposed to extend this permission to other areas, as more studies could be published by as prestigious journal. Assessing the first paragraph of the Instructions to Authors of the ABCD: "(...) is responsible for the publication of articles and clinical and experimental studies that contribute to the development of research, teaching and assistance in the field of surgical gastroenterology, clinical, endoscopic and other related issues. (...) ", I believe there is no conflict of interest in this request, since the mission of the journal is configured quite comprehensive.

We recently had the honor to correspond electronically with the Editor of the ABCD about a study related to Experimental Surgery, developed by Medicine students, which was very important in the formation of this group, it aroused huge interest of those involved and improved their school performance. However, this article may not be accepted due to, even though it was designed and guided by a general surgeon, traverse on topic that integrated the disciplines of Physiology, Histology, Pathology and animal testing was focused on kidney evaluation, making it impossible its publication in ABCD. Although the Editor has been extremely solicitous and kind to explain the refusal, and even suggest the publication in another journal of the same level, there was great disappointment of the students involved in the paper development.

Returning to the completion of Professor Fabio Gonçalves Ferreira1 Letter to the Editor: "Let us quote ourselves, let quote the ABCD" and the Editorial 2012 signed by Cleber Kruel and Osvaldo Malafaia (Arq Bras Cir Dig 2012; 25 (1): 1)5: "We will publish in ABCD! Let's quote the ABCD whenever we write a paper". Let me suggest: EXPAND THE ISSUES THAT MAY BE PUBLISHED IN ABCD!

Best regard. Pedro Luiz Squilacci Leme, Sao Paulo
